# The Gold(I) Complex with Plant Hormone Kinetin Shows Promising In Vitro Anticancer and PPARγ Properties

**DOI:** 10.3390/ijms24032293

**Published:** 2023-01-24

**Authors:** Zdeněk Trávníček, Ján Vančo, Jan Belza, Jan Hošek, Zdeněk Dvořák, René Lenobel, Igor Popa, Karel Šmejkal, Pavel Uhrin

**Affiliations:** 1 Czech Advanced Technology and Research Institute, Regional Centre of Advanced Technologies and Materials, Palacký University, Šlechtitelů 27, 77900 Olomouc, Czech Republic; 2 Department of Cell Biology and Genetics, Faculty of Science, Palacký University, Šlechtitelů 27, 78371 Olomouc, Czech Republic; 3 Laboratory of Growth Regulators, Institute of Experimental Botany of the Czech Academy of Sciences, Faculty of Science, Palacký University, Šlechtitelů 27, 78371 Olomouc, Czech Republic; 4 Department of Natural Drugs, Faculty of Pharmacy, Masaryk University, Palackého tř. 1946/1, 61242 Brno, Czech Republic; 5 Institute of Vascular Biology and Thrombosis Research, Center for Physiology and Pharmacology, Medical University of Vienna, Schwarzspanierstrasse 17, 1090 Vienna, Austria

**Keywords:** gold(I) complex, kinetin, anticancer, anti-inflammatory, in vitro, PPAR, cell cycle, apoptosis, ROS

## Abstract

Motivated by the clinical success of gold(I) metallotherapeutic *Auranofin* in the effective treatment of both inflammatory and cancer diseases, we decided to prepare, characterize, and further study the [Au(kin)(PPh_3_)] complex (**1**), where Hkin = kinetin, 6-furfuryladenine, for its in vitro anti-cancer and anti-inflammatory activities. The results revealed that the complex (**1**) had significant in vitro cytotoxicity against human cancer cell lines (A2780, A2780R, PC-3, 22Rv1, and THP-1), with IC_50_ ≈ 1–5 μM, which was even significantly better than that for the conventional platinum-based drug *Cisplatin* while comparable with *Auranofin*. Although its ability to inhibit transcription factor NF-κB activity did not exceed the comparative drug *Auranofin*, it has been found that it is able to positively influence peroxisome-proliferator-activated receptor-gamma (PPARγ), and as a consequence of this to have the impact of moderating/reducing inflammation. The cellular effects of the complex (**1**) in A2780 cancer cells were also investigated by cell cycle analysis, induction of apoptosis, intracellular ROS production, activation of caspases 3/7 and disruption of mitochondrial membrane potential, and shotgun proteomic analysis. Proteomic analysis of R2780 cells treated with complex (**1**) and starting compounds revealed possible different places of the effect of the studied compounds. Moreover, the time-dependent cellular accumulation of copper was studied by means of the mass spectrometry study with the aim of exploring the possible mechanisms responsible for its biological effects.

## 1. Introduction

**Figure 1:**
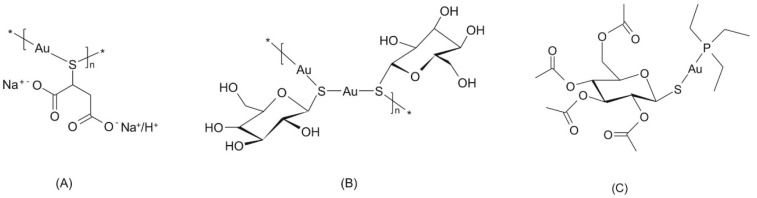
The structural formulas of three major gold-containing pharmacotherapeutics: natrium-aurothiomalate (**A**), aurothioglucose (**B**) and *Auranofin* (**C**). The asterisk symbols (*) mark the sites where the head-to-tail coupling of the monomeric units to the polymeric chain occurs.

Gold(I) complexes form an important group of compounds in the field of anti-inflammation therapy because they may represent a possible alternative to world-wide useable drugs such as sodium salt of 2-mercaptobutandiolate gold(I) (trivial title: polymeric natrium-aurothiomalate of a sum formula [C_4_H_(5−x)_O_4_SAuNa_x_]_n_, where x = 1 − 2), which represents a mixture of disodium and sodium salts (ATC code M01CB01, CAS: 12244-57-4) of a general formula (Figure 1A); polymeric [((2*S*,3*S*,4*R*,5*S*)-3,4,5-trihydroxy-6-(hydroxymethyl)-oxane-2-thiolate)gold(I)]_n_ complex (trivial title: aurothioglucose, ATC code M01CB04; CAS: 12192-57-3) of a general formula (Figure 1B); or [3,4,5-triacetyloxy-6-(acetyloxymethyl)-oxane-2-thiolate-(triethylphosphine)gold(I)] complex (trivial title: *Auranofin*, ATC code M01CB03; CAS: 34031-32-8) of a general formula (Figure 1C).

The positive biological features of *Auranofin* can not only be associated with its anti-inflammatory action related to its utilization in the treatment of rheumatoid arthritis [1] but also with its anticancer [2,3,4], antimicrobial [5], antibacterial [6,7], and antiparasitic [8] features. To date, many types of gold-complexes have been prepared and studied for the biological effects mentioned above and some of them revealed very promising results at both in vitro and in vivo levels. Among them, *Auranofin* analogues containing *N*-heterocyclic carbene (NHC) ligands play an important role. For instance, Au-NHC complexes of the composition [Au(NHC)Cl] and/or [Au(NHC)Cl]^+^ show very good in vitro cytotoxicity against various human cancer cell lines [9] or antibacterial [10,11] activity. Recent advances in Au complexes with various types of biological properties were reviewed by M. Mora et al. [12], by Y. Lu et al. [13], and by J. Zhang et al. [14]. In addition to Au-NHC complexes, the gold complexes with alkynyl [15] or diphenylphosphine [16] ligands were reviewed for their good anticancer effects.

The title complex (**1**) has been chosen by us as a suitable candidate for preparation and deeper biological studies owing to previous screening of anticancer and anti-inflammatory effects of gold(I) complexes containing variously substituted derivatives of 6-benzylaminopurine (HL) of the composition [Au(PPh_3_)(L)], which revealed promising in vitro anti-inflammatory features because they exhibit a strong ability to reduce the production of pro-inflammatory cytokines TNF-α, IL-1β, and HMGB1 without influence on the secretion of anti-inflammatory cytokine IL-1RA in the LPS-activated macrophages [17,18]. Some of these complexes showed comparable or even better anti-inflammatory effects as compared to a reference gold(I)-based drug *Auranofin*. Moreover, the complexes of a similar composition [Au(PPh_3_)(L1)] and [Au(PPh_3_)(L2)], containing hypoxanthine (HL1)- and deazahypoxanthine (HL2)-derived ligands, respectively, also showed significant in vitro anticancer activity against human cancer cell lines (e.g., A2780, A2780R, MCF-7, HOS, A549, G361) besides auspicious in vitro anti-inflammatory activity [19,20]. We wondered what would happen if kinetin (Hkin), as another nature-inspired ligand, is utilized instead of the above-mentioned ones in this structural type of complexes. Moreover, this approach could identify more effective potentially therapeutically useful compounds possessing both anti-inflammatory and antiproliferative activities. There is only scarce relevant information regarding the effectiveness of gold(I) complexes of this type in cell-based assays, selectivity of these compounds, and the details about their possible mechanism of action. The motivation for this study was associated with our efforts to prepare a compound a showing multimodal biological effect regarding its both anticancer (i.e., products of hydrolytic dissociation of the original complex could be responsible for the final cytotoxicity) and anti-inflammatory action (i.e., the complex could reveal dual anti-NF-κB and pro-PPARγ activities). Thus, the [Au(kin)(PPh_3_)] (**1**) complex was prepared, thoroughly characterized by elemental analysis; multinuclear ^1^H, ^13^C, ^15^N, and ^31^P NMR and FTIR spectral analyses; and mass spectrometry, with the thermal stability of the complex studied by TG/DSC analysis and the molecular and crystal structure of the complex determined by single crystal X-ray analysis. Further, the in vitro anticancer and anti-inflammatory activities were studied using the cell-based methods.

## 2. Results and Discussion

### 2.1. General Characterization

**Scheme 1:**
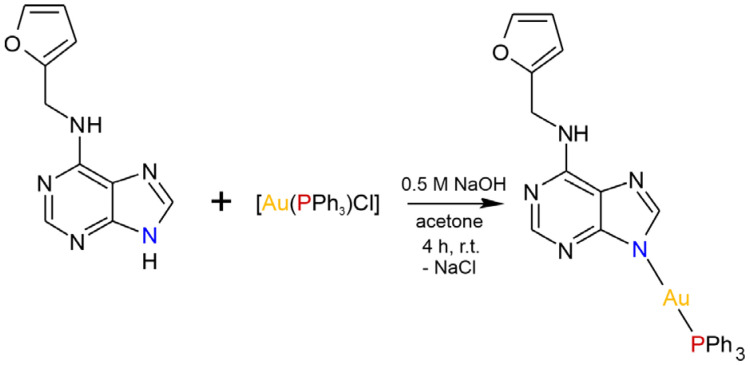
The reaction pathway leading to [Au(kin)(PPh_3_)] (**1**).

The [Au(kin)(PPh_3_)] complex (**1**) was synthesized using a reaction pathway depicted in Scheme 1. Firstly, it was characterized by elemental analysis; multinuclear ^1^H, ^13^C, ^15^N, and ^31^P NMR spectral data; and mass spectrometry, which confirmed not only the composition of the compound but also its sufficient purity. The CHN analysis revealed discrepancy of the % contents of C, H, and N elements not higher than 0.4%. The coordination of kinetin to the gold(I) atom through the N9 atom can be clearly seen from the values of coordination shifts for the HC^8^ proton (Δ*δ*_(**1**)-HKin_ = −0.89 ppm), C4 and C8 carbons (Δ*δ*_(**1**)-HKin_ = 6.55 ppm, and Δ*δ*_(**1**)-HKin_ = 8.79 ppm, respectively), and N9 nitrogen (Δ*δ*_(**1**)-HKin_ = 46.97 ppm). Besides this, the coordination of PPh_3_ to Au(I) through the P atom can be deduced from the coordination shift value of Δ*δ*_(**1**)-PPh3_ = 37.90 ppm. The atoms labelling based on the interpretation of NMR data is shown in Figure S1 in the Supplementary Materials. The complex (**1**) was subsequently studied using infrared spectroscopy, ESI+ mass spectrometry (see Figure S2 in the Supplementary Materials), and thermal analysis (see Figure S3 in the Supplementary Materials). The infrared spectra of (**1**) revealed medium-to-weak intensive peaks at 3240, 3119, and 2914 cm^−1^ that can be attributable to the stretching vibrations of ν(N–H), ν(C–H)_ar_, and ν(CH_2_), respectively. The peak of high intensity observed at 1609 cm^−1^ is assignable to ν(C–N)_ar_ vibrations, while the peaks of the ring ν(C–C)_ar_ vibrations were detected at 1561 and 1439 cm^−1^. The majority of the peaks observed within the interval of 660–900 cm^−1^ may be attributed to the skeletal vibrations of the purine moiety [21].

The ESI+ mass spectrum (see Figure S3 in the Supplementary Materials) showed peaks at *m/z* 696.16 and *m/z* 721.21 belonging to the {[Au(kin)(PPh_3_)]+Na}^+^ and [Au(PPh_3_)_2_]^+^ species, respectively.

The TG/DSC analyses of (**1**) revealed the thermal stability of the compound. It is necessary to note here that the sample used for thermal analysis was obtained similarly to the sample for X-ray structural study, i.e., by crystallization in the NMR tube from DMF after performing NMR experiments. As can be seen from Figure S4 in the Supplementary Materials, the complex (**1**) was stable up to around 85 °C, where it started to eliminate around 1/10 of the molecule of DMF as a crystal solvent molecule (calcd./exp.: 1.1/1.5%), accompanied by an endo-effect with the minimum at 90 °C. The thermal stability of an unsolvated form of the complex (**1**) lay in the interval of 95–206 °C. A very slight decrease in weight proceeded this in the interval of 206–245 °C, with minimum of the sharp endo-effect at around 238 °C. This can be connected with a combination of melting and decomposition of the complex (**1**). Then, the complex decomposed into three main waves accompanied by three exo-effects with maxima at around 281 °C, 353 °C, and 509 °C. The thermal decomposition was finished at around 600 °C.

### 2.2. Single Crystal X-ray Analysis

**Figure 2:**
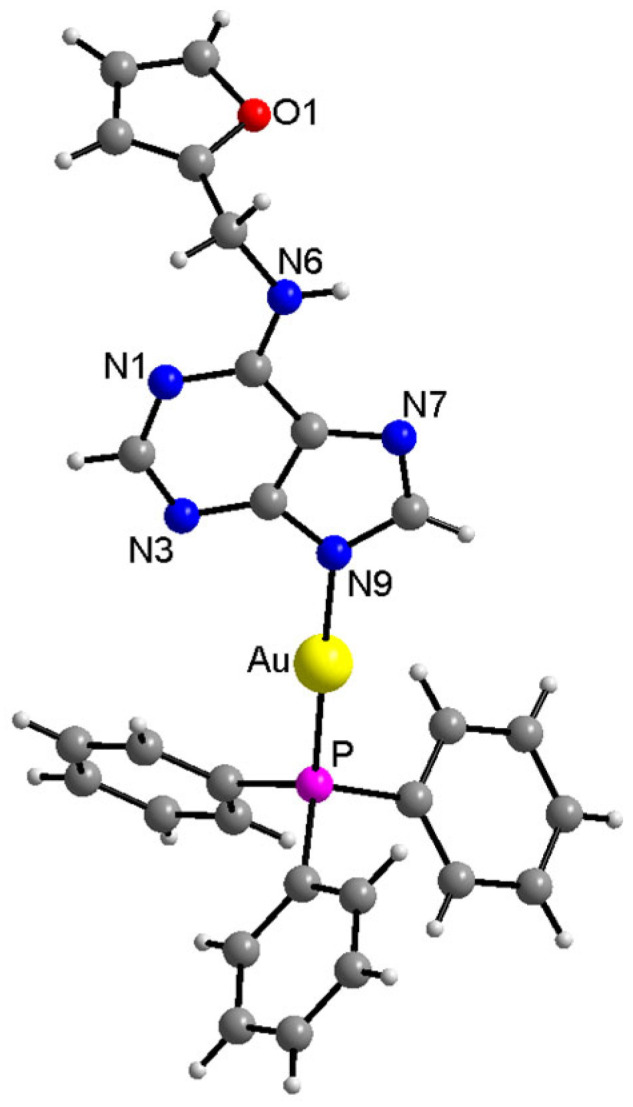
X-ray structure of [Au(kin)(PPh_3_)] (**1**). The labelling of the carbon (dark grey) and hydrogen (light grey) atoms is omitted for better clarity. The molecular graphics were generated in Diamond software (Crystal Impact, Bonn, Germany).

The structure of the complex (**1**) was unambiguously determined using a single crystal X-ray analysis, and it is depicted in Figure 2. The coordination mode of kinetin to the gold(I) atom proceeded via the N9 atom, as suggested by multinuclear NMR analysis. The selected interatomic parameters in the vicinity of the Au(I) atom, together with values of similar Au complexes (involving O-substituted-7-deazahypoxanthine (HL_1,2_) published previously [19,20]), are provided in Table 1. While the Au–P and Au–N9 bond lengths were nearly identical in all three compounds, the N9–Au–P angles differed significantly; however, this could be attributed to the different compositions of the N-donor ligands and different types and extents of hydrogen bonding in crystal packing. Two individual molecules of (**1**) were connected centrosymmetrically with the two N–H⋯N hydrogen bonds (see Figure S5 and Table S1 in the Supplementary Materials). The crystal structure was further stabilized by a variety of C–H⋯N, C–H⋯C, and C···C non-covalent contacts (see Figure S6 in the Supplementary Materials).

### 2.3. In Vitro Cytotoxicity Studies

In the first step, the in vitro cytotoxicity of [Au(kin)(PPh_3_)] (**1**), together with free kinetin, [Au(PPh_3_)Cl], *Auranofin*, and *Cisplatin* for comparative purposes, was determined on A2780, A2780R, and PC-3 human cancer cell lines. The results, summarized in Table 2, revealed a significant in vitro anti-proliferative effect of the complex (**1**), which markedly exceeded the activity of the commonly used cytostatic drug *Cisplatin*. However, on the other hand, the IC_50_ values of (**1**) were comparable with those determined for [Au(PPh_3_)Cl] and *Auranofin*, which may point out that the final cytotoxicity of these three compounds could be associated primarily with the formation of the [Au(PR_3_)]^+^ and/or [Au(PR_3_)_2_]^+^ species, as can be concluded from the results of the mass spectrometry experiments (see Figure S3 and [17,18,19,20]). The values of IC_50_ for kinetin were unable to be determined within the concentration range of 0.01–25 μM, and thus it can be expressed in the form of >25 μM. This finding is in accordance with literature data, where the in vitro cytotoxicity of kinetin on MCF-7 cells was determined at the IC_50_ = 52 µM [22]. Unfortunately, this fact undermined our original hypothesis that the dual effect of the [Au(PR_3_)]^+^ (and/or [Au(PR_3_)_2_]^+^) and free kinetin species should be probably responsible for the final cytotoxicity of (**1**). Nonetheless, we decided to extend the number of human cancer cells for the study, and thus the complex (**1**) was consequently studied on 22Rv1, THP-1, and LS180 (see Table 2) cells. The obtained results showed a strong anti-proliferative effect on THP-1 cells, while the IC_50_ value was unable to be determined from the dose–response curves within the concentration range of 0.01–10 μM. However, the complex (**1**) revealed good selectivity in connection with a value of its selectivity index (SI, which represents IC_50_ for normal cell line/IC_50_ for cancerous cell line), which was higher than 2. The SI value for *Auranofin* was around 1 in the case of all cancer cell lines, and thus its selectivity was negligible. 

### 2.4. Time-Dependent In Vitro Cytotoxicity and Cellular Uptake of Gold Complexes in A2780 Cells

In order to more precisely understand the dynamics of the antiproliferative effect of the title gold(I) complex, containing kinetin ligand, on A2780 cells, time-dependent experiments were performed using three incubation times, namely, 24 h, 48 h, and 72 h (see Table 3). Similarly to the results of in vitro cytotoxicity after 24 h incubation, the [Au(kin)(PPh_3_)] (**1**) and [Au(PPh_3_)Cl] complexes showed comparable effectiveness also after 48 h and 72 h incubation time with insignificant changes in IC_50_ values at around 3 µM levels. On the other hand, the reference drug *Auranofin* showed a gradual increase in antiproliferative activity over the increasing incubation time.

**Figure 3:**
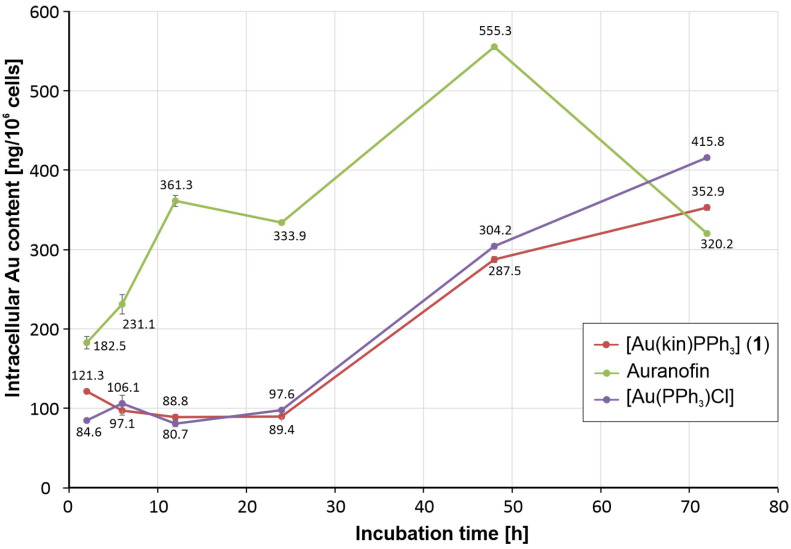
The intracellular gold concentration levels caused by the incubation of [Au(kin)(PPh_3_)] (**1**), [Au(PPh_3_)Cl], and *Auranofin* with A2780 cells after 2 h, 6 h, 12 h, 24 h, 48 h, and 72 h. The results were obtained by ICP-MS analyses of three parallel samples and are shown as mean values with error bars corresponding to the standard deviation values of the gold concentration expressed in ng of gold per 10^6^ cells.

The activity pattern observed by the time-dependent cytotoxicity experiments could be elucidated by the differences in cellular uptake of individual gold complexes into A2780 cells. As shown in Figure 3, the gradual increase in the antiproliferative effect of *Auranofin* was clearly attributable to significantly increasing intracellular gold levels, which from 2 h to 48 h of incubation significantly exceeded those observed for [Au(kin)(PPh_3_)] and [Au(PPh_3_)Cl] complexes up to 4 times excess after 12 h of incubation with A2780 cells. Only after 72 h of incubation were the intracellular gold levels induced by all three gold complexes under study at the comparable intracellular concentrations. The observed concentration patterns clearly correlated with the results of time-dependent cytotoxicity assays.

### 2.5. Modification of A2780 Cell Cycle by the Gold(I) Complexes

**Figure 4:**
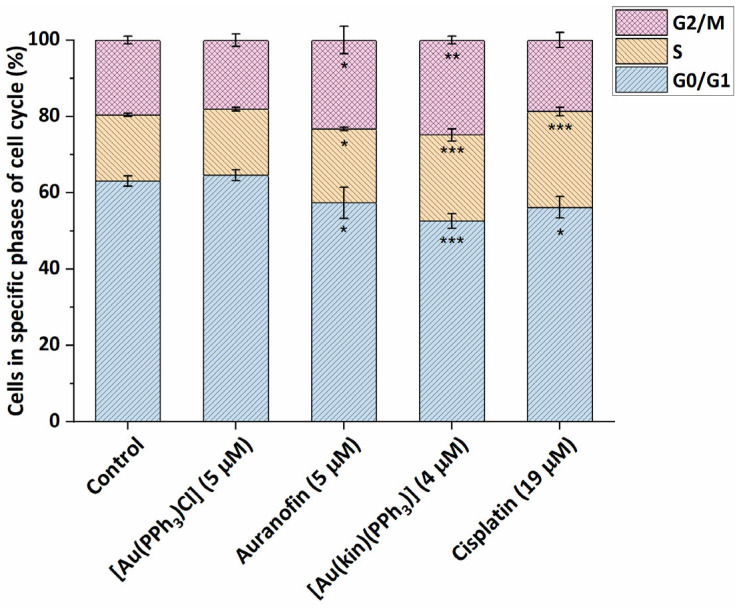
Modifications of the cell cycle of A2780 cells caused by the 24 h incubation of (**1**), starting complex [Au(PPh_3_)Cl], and reference drugs *Auranofin* and *Cisplatin*, applied at half-cytotoxic concentration levels. The statistical significance was considered at the following levels: * *p* < 0.05, ** *p* < 0.01, *** *p* < 0.001 with respect to the untreated control group.

In addition to the cytotoxicity studies, we strived to understand more deeply the cellular and molecular mechanisms involved in the biological action of the studied gold(I) complexes. In the first stage, the effects of the complexes on one type of the susceptible cancer cells, namely, A2780 cells, was studied. The gold(I) complexes, (**1**), starting complex [Au(PPh_3_)Cl], reference drug *Auranofin*, and platinum-based drug *Cisplatin* were applied at half-cytotoxic concentration and incubated for 24 h. The starting complex [Au(PPh_3_)Cl] showed practically no effect on the cell cycle of A2780 cells, i.e., most of the cells resided in a quiescent state at the G0/G1 phase (see Figure 4). On the other hand, both gold(I) complexes (**1**) and *Auranofin*, as well as *Cisplatin,* caused a significant decrease in the number of cells in the G0/G1 phase. The gold(I) complexes (**1**) and *Auranofin* significantly increased the population of cells in G2/M (in 24.8 ± 1.0% for (**1**), and 23.3 ± 3.6% for *Auranofin*) and S phase (in 22.6 ± 1.6% for (**1**), and 19.4 ± 0.5% for *Auranofin*), while the effect of (**1**) was in both cases stronger than that of *Auranofin*. These cellular effects on the cell cycle of cancer cells are consistent with the patterns of biological activities published for other gold(I/III) complexes elsewhere, for example, gold(III) complex [Au(tpy)Cl(2-TU)], where tpy represents 2-(*p*-tolyl)pyridine and 2-TU represents 2-thiouracil, which in addition to its high antiproliferative effect in vitro against Caco-2 cells (IC_50_ = 0.51 ± 0.28 µM) caused the specific G2/M arrest of human-enterocyte-like Caco-2/TC7 cells in around 30% of cells [23]; or the gold(I)-chlorido complex involving the chiral (*R*,*R*)-(−)-2,3-bis(*t*-butylmethylphosphino)quinoxaline ligand, which caused the G2/M cell phase arrest in OVCAR8 cells after 24 h incubation in around 21% cells and S-phase arrest in around 36% of cells [24]; or the diethyldithiocarbamato-[(1,1′-biphenyl-2-yl)di-*tert*-butylphosphine] gold(I) complex, which caused G2/M arrest in A2780 cells in around 30% of cells at 23 nM concentration [25]. As expected, *Cisplatin* arrested a significant number of cells in S-phase, which is consistent with its mechanism of action (dominantly the covalent modifications of DNA) [26,27].

### 2.6. Induction of Cell Death in A2780 Cells

**Figure 5:**
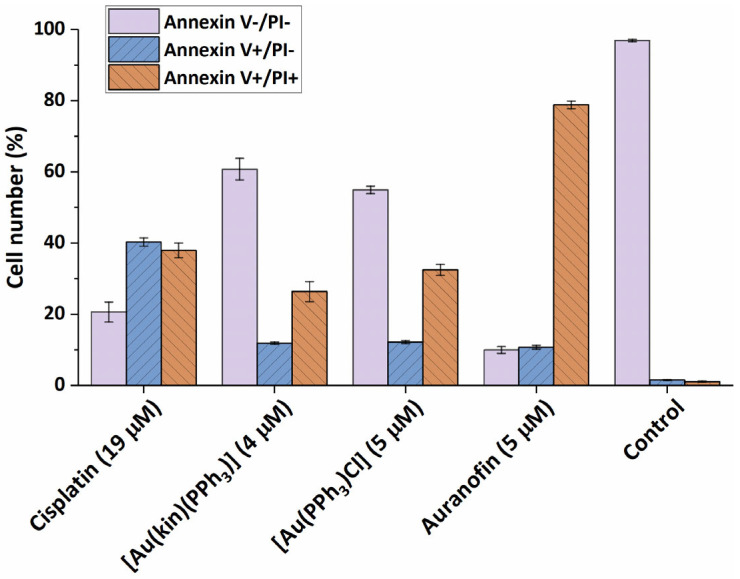
The ability of (**1**) and the reference gold(I) complexes [Au(PPh_3_)Cl], *Auranofin*, and a reference drug *Cisplatin* to cause the transformation of intact A2780 cells (Annexin V-/PI-) towards early apoptosis (Annexin V+/PI-) and late-stage apoptosis (Annexin V+/PI+) after 24 h of incubation with half-cytotoxic concentrations of the compounds. The changes in all groups were found to be significant at the *p* < 0.0001 level with respect to the untreated control group.

**Figure 6:**
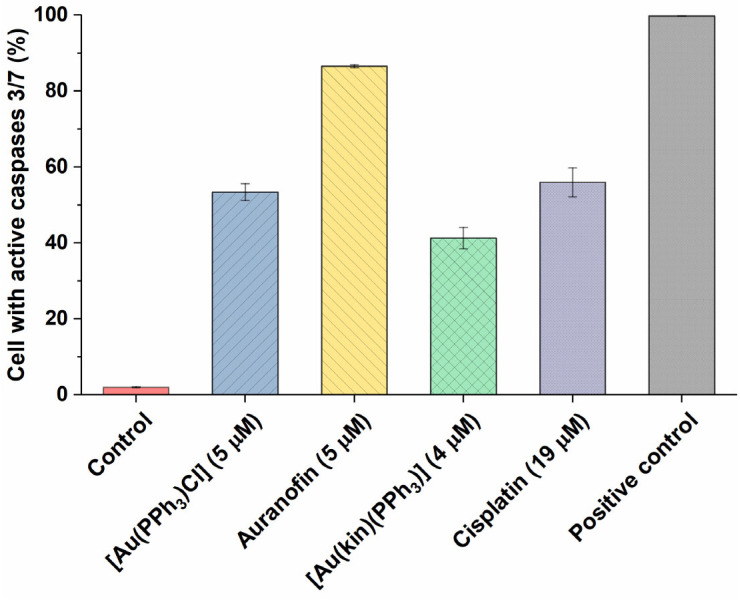
The ability of (**1**) and the reference gold(I) complexes [Au(PPh_3_)Cl], *Auranofin*, and a reference drug *Cisplatin* to activate the executioner caspases 3/7 in A2780 cells after 24 h of incubation with half-cytotoxic concentrations of the compounds. The changes in all groups were found to be significant at the *p* < 0.0001 level with respect to the untreated control group. The positive control represents the untreated cells heated to 60 °C for 10 min.

**Figure 7:**
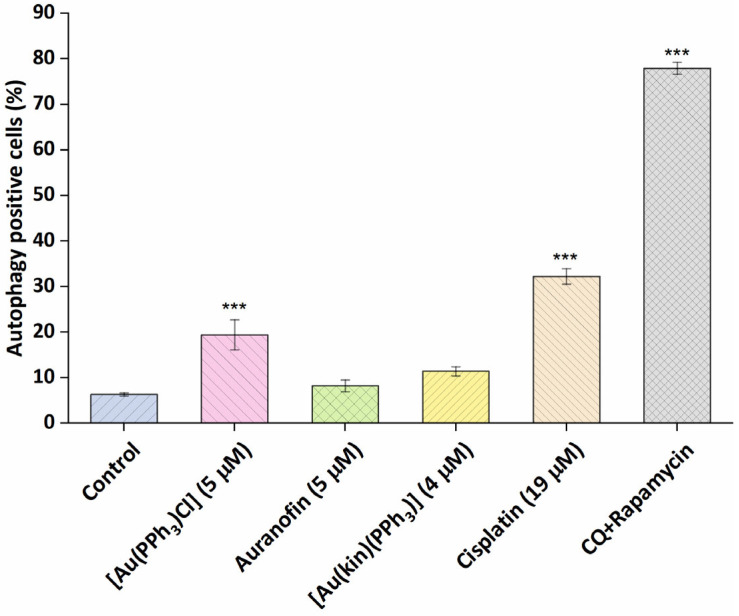
The effectiveness of (**1**), the starting complex [Au(PPh_3_)Cl], and the reference drugs *Auranofin* and *Cisplatin* to induce autophagy of A2780 cells after 24 h of incubation with half-cytotoxic concentrations of the compounds. The statistical significance was considered at the *** *p* < 0.001 level with respect to the untreated control group. The positive control represents the cells treated with the mixture of chloroquine and rapamycin (CQ + rapamycin), a known strong inducer of autophagy.

To better pinpoint the mechanism underlying the cytotoxicity of gold(I) complexes in A2780 cells, the flow cytometry experiments involving the Annexin V/PI fluorescent staining for the detection of cellular structure destruction (usual for apoptosis) (see Figure 5) and the activation of executioner caspases 3/7 (see Figure 6), specific for late-stage apoptosis and induction of autophagy (see Figure 7), representing the normal reaction of cells on stressor stimuli, were performed on A2780 cells after 24 h incubation with half-cytotoxic concentrations of the tested gold(I) compounds and compared with the effects of the platinum-based drug *Cisplatin* and/or the specific positive control.

Both (**1**) and starting complex [Au(PPh_3_)Cl] showed similar effect on A2780 cell death, leading to the induction of early stage apoptosis in around 10% of the cells and late stage apoptosis in around 30% of cells after 24 h incubation. On the other hand, the reference drug *Cisplatin* caused the reversion of the cells to early and late-stage apoptosis in around 40% of cells, and *Auranofin* showed a strong proapoptotic effect, leading to around 80% of A2780 cells in late-stage apoptosis after 24 h.

In continuation of the Annexin V/PI staining, the determination of the number of cells with active executioner caspases 3/7 followed. The obtained results roughly correlated with the effectiveness of the compounds to induce late-stage apoptosis, rendering the *Auranofin* as the most effective gold(I) complex. Nevertheless, [Au(PPh_3_)Cl] and *Cisplatin* (in around 50% of cells) and (**1**) in around 40% of cells were also effective activators of downstream caspases 3/7.

It is a known fact that the metabolic stressors, such as *Cisplatin* [28] can provoke autophagy, a tissue-protective process preventing harm to a wider number of cells or the whole organism. It is also considered one of the mechanisms of intrinsic resistance towards deleterious xenobiotics. In our experiments (Figure 7), *Cisplatin* and the starting complex [Au(PPh_3_)Cl] significantly induced the autophagy in A2780 cells. *Auranofin*, in contrast to previously reported autophagy induction in HT1080 fibroma cells acting through the degradation of IRF3 and impeding its transcriptional and proapoptotic activities [29] at 5 µM concentration, together with complex (**1**), was not shown to cause metabolic stress strong enough to induce the autophagy in the A2780 cancerous cells after 24 h incubation.

### 2.7. The Effect of Gold(I) Complexes on Intracellular ROS Levels and Damage to the Mitochondrial Membranes

One of the main postulated mechanisms of action of gold(I) complexes is the inhibition of the major antioxidant selenium-containing enzymes (mainly thioredoxin reductase), leading to the disruption of the intracellular thiol-recovery systems, depletion of reduced glutathione, and oxidative stress [30]. The next consequence of this process is the dysfunction of mitochondrial metabolism, destruction of mitochondrial membranes, and induction of the intrinsic apoptotic pathway [31]. Nevertheless, there are some reports [32] that point out the variability of biological response between different types of cancer cells (susceptible leukemia vs. solid tumor cells) towards the pro-oxidant action of *Auranofin*. Our results showed relatively high variability of biological responses for the intracellular ROS levels (see Figure S7 in the Supplementary Materials) and superoxide levels (see Figure S8 in the Supplementary Materials). However, in all cases, both the tested gold(I) complexes and *Cisplatin* did not cause a significant increase in the intracellular oxidative stress, even if co-incubated with pro-oxidant pyocyanin.

**Figure 8:**
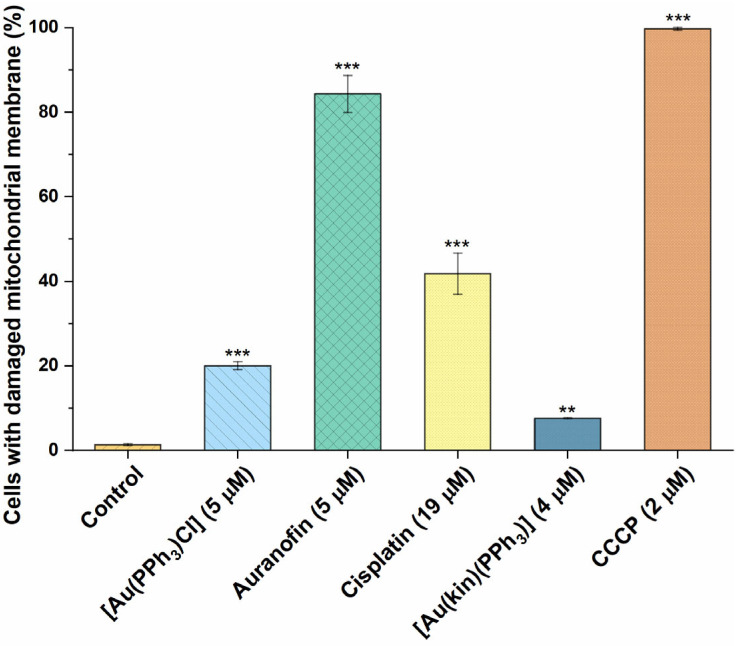
The demonstration of damage to mitochondrial membranes in A2780 cells after 24 h of incubation with half-cytotoxic concentrations caused by (**1**), the starting complex [Au(PPh_3_)Cl], and the reference drugs *Auranofin* and *Cisplatin*. The statistical significance was considered at the following levels: ** *p* < 0.01, *** *p* < 0.001 with respect to the untreated control group. The positive control represents the cells treated with carbonyl cyanide chlorophenylhydrazone (CCCP), a well-documented agent causing the mitochondrial function disruption.

On the other hand, the 24 h incubation of A2780 cells with *Auranofin* led to extreme changes in the permeability of the mitochondrial membrane and, consequently, the loss of the membrane potential in more than 80% of cells (see Figure 8). This clearly correlates with the known ability of *Auranofin* to induce the intrinsic pathway of apoptosis. Significant changes in mitochondrial membrane permeability were also caused by *Cisplatin* and [Au(PPh_3_)Cl], leading to the conclusion that the above-mentioned mechanism of action could attribute to the cytotoxicity potential of these agents. The complex (**1**) was clearly the least effective—the moderation of mitochondrial metabolism caused by this complex could be attributed to its biological effect (cytotoxicity against cancer cells), and therefore its mechanism of action is clearly multifactorial.

### 2.8. The Effect on NF-κB Activity Induced by LPS and PPARγ

To determine the highest non-toxic concentration of the tested compounds, their influence on cell viability was determined for THP1-Blue™ NF-κB cells incubated in serum-free medium for 24 h (for further details, see Table S2 in the Supplementary Materials). The cytotoxicities of (**1**) were comparable, regardless of whether the THP-1 cells were grown in complete medium (i.e., containing FBS), or without the FBS in the culture medium. This indicates that the presence of serum proteins in the media does not influence the biological effect of this complex. The observed effect of (**1**) on cell viability was similar to other previously published triphenylphosphine–gold(I) complexes containing adenine-derived ligands [17], i.e., the tested gold(I) complexes possessed a less cytotoxic effect than *Auranofin*. For further analyses of anti-inflammatory potential, the concentration of 100 nM was selected as safe and non-toxic.

**Figure 9:**
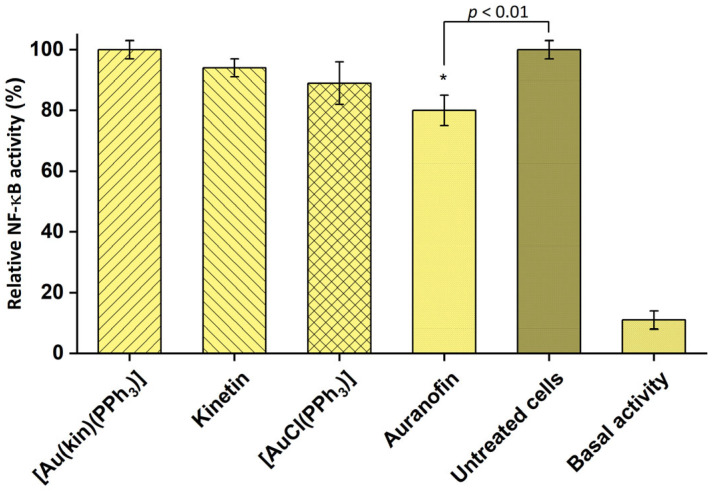
Effect of the tested compounds on NF-κB activity in THP1-Blue™ NF-κB monocytes. Effect of 1 h pre-treatment on LPS-stimulated NF-κB activity detected after 24 h. The statistical significance was considered at the * *p* < 0.01 level as compared to the vehicle (DMF) group. Data are shown as means ± SEM.

*Auranofin* is known for its strong anti-inflammatory potential [33], and indeed it was the only gold(I) complex able to inhibit the activity of pro-inflammatory transcription factor NF-κB also in our case. On the contrary, complex (**1**) did not show any effect on the activity of NF-κB, even though the ligand kinetin and starting complex [Au(PPh_3_)Cl] possessed some activity (see Figure 9).

**Figure 10:**
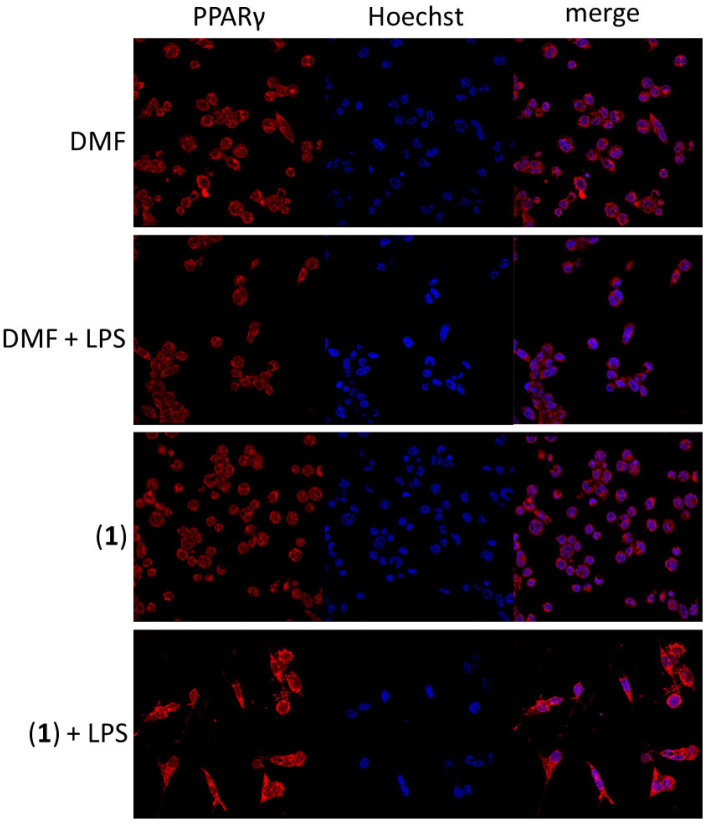
The effect of (**1**) on the amount and intracellular localization of PPARγ in THP1-Blue™ NF-κB macrophages after 1 h pre-treatment. The visual data were collected using a Leica TCS SP8 MP confocal microscope (Leica Microsystems, Mannheim, Germany).

There are some clues that gold-based complexes could interact with peroxisome-proliferator-activated receptor-gamma (PPARγ) [34] and thus possess the anti-inflammatory effect in target cells. By means of immunocytochemistry assay, we revealed the potential of (**1**) to slightly increase the amount of PPARγ in LPS-stimulated THP1-Blue™ NF-κB macrophages (see Figure 10). On the contrary, both kinetin or [Au(PPh_3_)Cl] did not show this effect (see Figure S9 in the Supplementary Materials). This effect on PPARγ in such a way was described in gold(I) complexes for the first time. It could point out a new potential anti-inflammatory mechanism of action of complex (**1**).

### 2.9. Proteomic Analysis of Gold(I) Complexes [Au(kin)(PPh_3_)] *(**1**)* and [Au(PPh_3_)Cl] on Changes in the Proteome of R2780 Cells

**Figure 11:**
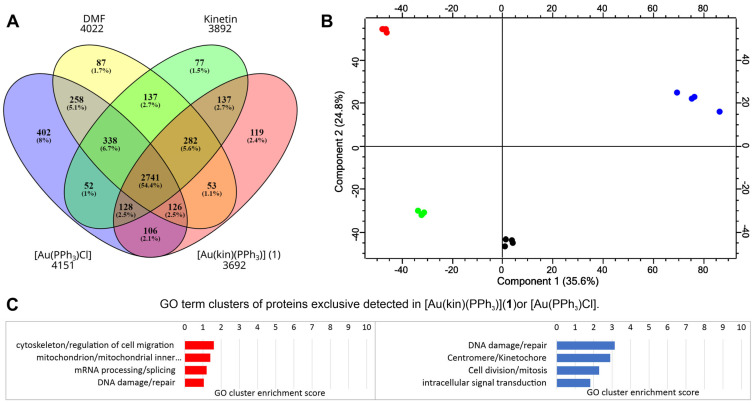
The summary of proteomic results of the R2780 cells treated with the gold complexes [Au(kin)(PPh_3_)] (**1**) and [Au(PPh_3_)Cl], and reference samples kinetin and dimethylformamide (DMF). (**A**) An overview of the results of all four samples. The numbers below the samples’ labels represent the total number of identified proteins for each compound. (**B**) The PCA plot of the analyzed samples. Red color belongs to [Au(kin)(PPh_3_)] (**1**), blue color to [Au(PPh_3_)Cl], green color to the reference sample kinetin, and black color to the reference sample DMF. (**C**) A summary of the bioinformatic analysis of the enrichment of GO terms of the protein exclusively identified in the sample treated with the gold complexes. The red chart represents the sample treated with [Au(kin)(PPh_3_)] (**1**), and the blue chart represents the sample treated with [Au(PPh_3_)Cl]. Four technical replicates represent each biological sample. A Venn diagram was created by the tool Oliveros, J.C. (2007–2015) Venny, an interactive tool for comparing lists with Venn diagrams: https://bioinfogp.cnb.csic.es/tools/venny/index.html (accessed on 28 April 2022). The PCA plot was created by the Perseus tool v. 2.0.7.0.

To evaluate the effects of treatments of the gold(I) complexes [Au(kin)(PPh_3_)] (**1**) and [Au(PPh_3_)Cl] on the proteome of R2780 cells, the shotgun proteomic analysis was conducted. The summary of collected identification data is summarized in Figure 11. In all samples, we identified 5202 proteins and 21,578 peptides. The distribution of identified proteins among samples is depicted in the Venn diagram shown in Figure 11A. The same set of identified proteins in all samples comprised 53% of all identified proteins, but also there were proteins exclusively identified in each particular sample. Exclusively identified proteins 119 and 402 in the samples treated with the [Au(kin)(PPh3)] (**1**) and [Au(PPh3)Cl], respectively, were analyzed using DAVID bioinformatic tools to evaluate the enrichment of GO terms. The most enriched GO term categories are depicted in Figure 11C. The complex [Au(PPh_3_)Cl] had a strong effect on the activation of proteins responsible for DNA repair after DNA damage and also affected cell division and mitosis. On the other hand, the complex [Au(kin)(PPh_3_)] (**1**) affected activation of proteins responsible for cell cytoskeleton/regulation of cell migration and mitochondrial inner membrane proteins.

**Figure 12:**
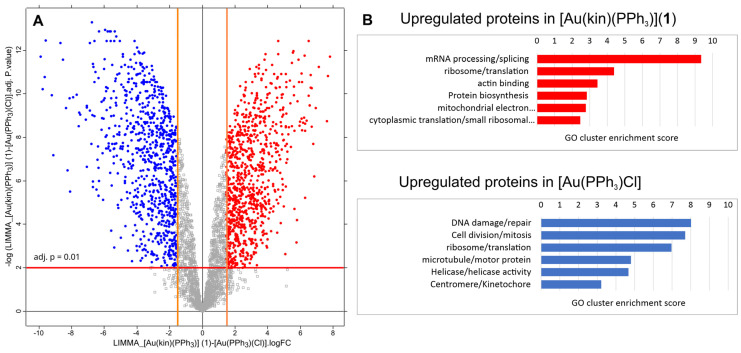
(**A**) The volcano plot of differences at protein levels between samples treated with [Au(kin)(PPh_3_)] (**1**) versus [Au(PPh_3_)Cl]. The lines in the chart define the stringency of detecting upregulated proteins. The red line represents the adj. *p*-value at 0.01. The orange lines define the minimal limits of protein fold-change to be taken as upregulated. They were set to log2(FC) equal to 1.58, which states that the change of the protein level between differentially treated samples must be at least three times higher. Red dots represent the proteins upregulated in the sample treated with the complex [Au(kin)(PPh_3_)] (**1**). Conversely, the blue dots represent the proteins upregulated in the sample treated with the complex [Au(PPh_3_)Cl]. (**B**) Summary of the enrichment of GO terms for upregulated proteins in each sample. The volcano plot was created by the Perseus tool v. 2.0.7.0.

The PCA plot shown in Figure 12B displays the significant differences among differentially treated samples. The difference between proteomes of kinetin (green-filled circles)- and DMF (black-filled circles)-treated samples were minimal, which could be expected because the kinetin or DMF did not have a cytotoxic effect on the R2780 cell at low concentration. On the other hand, the treatment of both the complexes [Au(kin)(PPh_3_)] (**1**) (red-filled circles) and [Au(PPh_3_)Cl] (blue-filled circles) had a dramatic effect on the R2780 cells, and they also highly differed between themselves. To deeply evaluate differences between proteomes of R2780 cells treated with both gold complexes [Au(kin)(PPh_3_)] (**1**) and [Au(PPh_3_)Cl], the quantitative analysis of proteomic data was carried out.

Figure 12 summarizes the quantitative analysis of differences between samples treated by [Au(kin)(PPh_3_)] (**1**) versus [Au(PPh_3_)Cl]. In Figure 12A, the volcano plot is displayed and shows relative fold changes (FC) of protein levels between the samples [Au(kin)(PPh_3_)] (**1**) versus [Au(PPh_3_)Cl]. The red dots represent 854 proteins upregulated in the sample treated with [Au(kin)(PPh_3_)] (**1**) with FC greater or equal to 3. In contrast, the blue dots represent 821 proteins upregulated in the sample treated with [Au(PPh_3_)Cl]. These two protein lists were analyzed with DAVID bioinformatic tools to estimate an enrichment of GO terms. The most enriched GO term categories are depicted in Figure 12B. Again, the complex [Au(PPh_3_)Cl] strongly affects proteins responsible for DNA damage/repair, cell division/mitosis, ribosome/translation, and other fields. The complex [Au(kin)(PPh_3_)] (**1**) exerts influences on proteins responsible for mRNA processing/splicing, ribosome/translation, actin binding, protein biosynthesis, and others.

## 3. Materials and Methods

### 3.1. General Methods Used for Characterization

The [Au(kin)(PPh_3_)] complex (**1**) was characterized using CHN elemental analyses (CHN analyser Flash Smart CHN, Thermo Scientific, Waltham, MA, USA), infrared spectroscopy (IR) performed using an ATR technique and in the range of 400–4000 cm^−1^ (Nicolet iS5 FT–IR, Thermo Nicolet), TG/DSC thermogravimetric analyses in the temperature range of 23 °C to 900 °C (Netzsch STA 449 F1 Jupiter^®^, Bayern, Germany), and mass spectrometry (MS) performed using the ESI+ technique (Bruker amaZon SL, Bruker, Billerica, MA, USA). ^1^H, ^13^C, ^15^N, and ^31^P NMR spectra were recorded using a JEOL JNM-ECA 600II (Tokyo, Japan) device on the DMF-*d*_7_ solutions at 300 K and single crystal X-ray analysis preformed using a D8 Quest diffractometer (Bruker, Billerica, MA, USA) at 293 K.

### 3.2. Materials

Chemicals and solvents were purchased from the commercial sources Sigma–Aldrich Co., (Burlington, MA, USA) and Acros Organics Co., (Geel, Fluka Co., and Lachema Co.) and were used without any further purification. Free kinetin was prepared by a slightly modified procedure described in the literature [35], and its composition and purity were checked by elemental analysis and NMR spectroscopy. [Au(PPh_3_)Cl] was synthesized as described in [36,37].

### 3.3. Preparation and Characterization of the Complex *(**1**)*

The complex (**1**) was prepared as follows: 0.5 mmol of [Au(PPh_3_)Cl] was dissolved in 10 mL of acetone, and 0.5 mmol of kinetin dissolved in 30 mL of acetone was added into the solution during stirring. Then, 0.5 mL of 0.5 M NaOH was added dropwise to the reaction mixture, which was stirred at room temperature for the next 4 h (see Scheme 1). After that, the NaCl that formed was filtered off, and colorless filtrate was to left stand to evaporate. After a few days, white-yellow solid formed, which was separated by filtration, washed with 2 × 5 mL of methanol and 5 mL of diethyl ether, and dried at 40 °C under an infrared lamp.

Complex (**1**): Anal. Calcd. for C_28_H_23_N_5_O_1_Au_1_P_1_ (Mr = 673.5): C, 49.9; H, 3.4; N, 10.4. Found: C, 49.9; H, 3.6; N, 10.2%. IR (KBr; cm^−1^): 466, 501, 546, 692, 713, 747, 811, 964, 997, 1009, 1103, 1151, 1210, 1263, 1315, 1382, 1439, 1472, 1561, 1609 ν(C–N)_ar_, 2914, 3030, 3119, 3164, 3420.

**^1^H**-NMR, *d_7_*-DMF, T = 25 °C, relative to TMS, *δ* (ppm), *J* (Hz): 8.16, 1H, *s*, HC^2^, Δ*δ*_(**1**)-HKin_ = −0.09; 7.93, 1H, *s*, HC^8^, Δ*δ*_(**1**)-HKin_ = −0.89; 7.76, 6H, *mm*, 7.5, HC^2,6; 2′,6′; 2″,6″ (PPh^_3_^)^ Δ*δ*_(**1**)-PPh3_ = 0.44; 7.71, 3H, *mm*, 7.5, HC^4,4′,4″ (PPh^_3_^)^, Δ*δ*_(**1**)-PPh3_ = 0.29; 7.68, 6H, *mm*, 7.5, HC^3,5; 3′,5′; 3″,5″ (PPh^_3_^)^, Δ*δ*_(**1**)-PPh3_ = 0.26; 7.54, 1H, *s*, HC^13^, Δ*δ*_(**1**)-HKin_ = −0.02; 7.43, 1H, *br*, HN^6^, Δ*δ*_(**1**)-HKin_ = −0.50; 6.36, 1H, *t*, 2.6, HC^12^, Δ*δ*_(**1**)-HKin_ = −0.02; 6.28, 1H, *d*, 2.6, HC^11^, Δ*δ*_(**1**)-HKin_ = −0.03; 4.88, 2H, *br*, HC^9^, Δ*δ*_(**1**)-HKin_ = 0.03.

**^13^C**-NMR, *d_7_*-DMF, T = 25 °C, relative to TMS, *δ* (ppm): 157.49 (C4), Δ*δ*_(**1**)-HKin_ = 6.55; 154.91 (C6), Δ*δ*_(**1**)-HKin_ = 0.61; 154.09 (C10), Δ*δ*_(**1**)-HKin_ = −1.17; 151.44 (C2), Δ*δ*_(**1**)-HKin_ = −1.73; 148.51 (C8), Δ*δ*_(**1**)-HKin_ = 8.79; 142.42 (C13), Δ*δ*_(**1**)-HKin_ = −0.17; 135.00, *d*, 13.7 (C 2,6; 2′,6′; 2″,6″), Δ*δ*_(**1**)-PPh3_ = 0.21; 133.09 (C4, 4′, 4″), Δ*δ*_(**1**)-PPh3_ = 2.99.; 130.42, *d*, 11.9 (C3,5; 3′,5′; 3″,5″), Δ*δ*_(**1**)-PPh3_ = 0.24; 129.50, *d*, 63.2 (C1,1′,1″), Δ*δ*_(**1**)-PPh3_ = −9.00; 120.97 (C5), Δ*δ*_(**1**)-HKin_ = 0.96; 111.01 (C12), Δ*δ*_(**1**)-HKin_ = −0.905; 107.02 (C11), Δ*δ*_(**1**)-HKin_ = −0.33; 37.85 (C9), Δ*δ*_(**1**)-HKin_ = 0.35.

**^15^N**-NMR, *d_7_*-DMF, T = 25 °C, relative to DMF (104.7), *δ* (ppm), *J* (Hz): 7.93, *s*, HC^8^/204.29 (h. int.), N9, Δ*δ*_(**1**)-HKin_ = 46.97; 8.16, *d*, 12.5, HC^2^/230.71 (l. int.), N1, Δ*δ*_(**1**)-HKin_ = 0.65; 8.16, *s*, HC^2^/237.93 (h. int.), N3, Δ*δ*_(**1**)-HKin_ = 6.15; 7.93, *s*, HC^8^/241.20 (h. int.), N7, Δ*δ*_(**1**)-HKin_ = −0.20; N6–no signal detected, h. int, and l. int—high and low intensity.

**^31^P**-NMR, *d_7_*-DMF, t = 25 °C, relative to H_3_PO_4_ (electronic), *δ* (ppm): 32.46, Δ*δ*_(**1**)-PPh3_ = 37.90, Δ*δ*_(**1**)-PPh3_ = –1.27.

### 3.4. Single-Crystal X-ray Diffraction Analysis

Single crystals of (**1**) suitable for single-crystal X-ray analysis were grown from DMF-*d_7_* solution of (**1**) in NMR tube after the realization of NMR experiments. The data were collected on a Bruker D8 QUEST diffractometer equipped with a PHOTON 100 CMOS detector using Mo-Kα radiation (*λ* = 0.71073 Å) at 293 K. The APEX3 software package [38] was used for data collection and reduction. The molecular structure was solved by direct methods (SHELXS) and refined by full-matrix least-squares procedure SHELXL (version 2014/7) [39]. Hydrogen atoms were found in the difference Fourier maps and refined using a rigid model, with C–H = 0.95 Å (CH)_ar_ and (CH), C–H = 0.99 Å (CH_2_), C–H = 0.98 Å (CH_3_) and O–H = 0.84 Å (OH), and with *U*_iso_(H) = 1.2*U*_eq_(CH, CH_2_, OH) and *U*_iso_(H) = 1.5*U*_eq_(CH_3_). The figures were drawn, and additional structural calculations were performed using DIAMOND [40] and MERCURY [41] software. Owing to a highly disordered unknown solvent molecule (a disordered DMF molecule with highest probability), a region of disordered electron density was treated using a SQUEEZE procedure [42] incorporated in PLATON software [43]. The crystal data and structure refinement details are given in Table 4.

### 3.5. In Vitro Cytotoxicity against Human Cancerous and Normal Cell Lines

The in vitro cytotoxicity of complex (**1**), and kinetin, [Au(PPh_3_)Cl], *Auranofin*, and *Cisplatin*, for comparative purposes, was determined by the MTT assay at various time of incubation (24, 48, and 72 h), as previously described in more detail [17,18,19,20]. The testing was performed on the ovarian carcinoma (A2780), *Cisplatin*-resistant ovarian carcinoma (A2780R), prostate adenocarcinoma (PC-3), prostate carcinoma (22Rv1), and leukemia monocytic (THP-1) and intestinal colon adenocarcinoma (LS180) human cell lines that were obtained from the ATCC collection of cell lines and cultivated according to the producer’s instructions. The reference normal cell line of human fetal fibroblasts (MRC-5) was obtained from the same commercial source and maintained according to the producer’s instructions. The half-maximal inhibitory concentrations (IC_50_) were calculated from dose–response curves by means of the GraphPad Prism 6 software (GraphPad Software, San Diego, CA, USA).

### 3.6. Cell Cycle Analysis

The A2780 cells (Sigma, 93112519-1VL) were cultivated according to the producer’s instructions and seeded at 10^4^ cells/well in 96 wells. After 24 h, the cells were treated with a 4 µM solution of complex (**1**) and cultivated for the next 24 h. Then, the cells were washed once with PBS (0.1 M, pH 7.4), and cell cycle analysis was performed using a BD Cycletest^TM^ Plus DNA kit (Becton Dickinson, Franklin Lakes, NJ, USA) according to the manufacturer’s protocol. The data were acquired using a BD FACSVerse flow cytometer (Becton Dickinson, USA) in three independent experiments, each performed in duplicate, while at least 5 × 10^3^ events were recorded for each sample.

### 3.7. Induction of Cell Death and Related Processes

The induction of cell death by complex (**1**) was studied by two methods in the A2780 cell line. Firstly, the induction of apoptosis was tested using an Annexin V-FITC/PI commercial kit (V13242, Thermo Fisher Scientific, USA). Second, caspase 3/7 activation was monitored using the CellEvent™ Caspase-3/7 Green Flow Cytometry Assay Kit (C10427, Thermo Fisher Scientific, USA). Both assays were performed according to the manufacturers’ protocols with one modification in the caspase induction assay: the use of CellEvent^TM^ Caspase-3/7 Green Detection Reagent was only for the detection of caspase 3/7 activation. The general procedure was as follows: 5 × 10^4^ cells per well were seeded in a 24-well cell culture plate, and after 24 h, the cells were treated with 4 µM solution of complex (**1**) and cultivated for next 24 h. The next day, the cells were washed once with PBS (0.1 M, pH 7.4) and trypsinized using 0.25% trypsin-EDTA (Gibco™, Waltham, MA, USA). The trypsin was blocked by adding the cell culture medium to a final volume of 500 µL, and the resulting cell suspensions were divided into two separate 250 µL aliquots. The first set of aliquots was used to evaluate the Annexin V-FITC/PI assay, whilst the second one was used for the caspase 3/7 activation assay. Both assays were performed using a BD FACSVerse flow cytometer (Becton Dickinson, USA) in three independent experiments, each performed in duplicate.

### 3.8. Induction of Autophagy

The induction of autophagy by complex (**1**) was studied using a CYTO-ID^®^ Autophagy Detection Kit 2.0 (ENZO, New York, NY, USA) according to the manufacturer’s protocol. Briefly, the A2780 cells were seeded in a 24-well cell culture plate at 5 × 10^4^ cells per well and cultivated for 24 h. The next day, the cells were treated with complex (**1**) at 4 µM. After a 24 h incubation period, the cells were washed once with PBS (0.1 M, pH 7.4), trypsinized using 0.25% trypsin-EDTA (Gibco™), and resuspended in a cell culture medium. Then, the cells were stained with CYTO-ID^®^ Green stain solution and incubated for 30 min in the dark. After the final wash with PBS, the cell suspensions were analyzed using the BD FACSVerse flow cytometer (Becton Dickinson, USA) in three separate experiments, while at least 10^4^ events were recorded for each sample prepared in duplicates. The cells incubated with the mixture of chloroquine (10 µM) and rapamycin (0.5 µM) for 18 h were used as a positive control.

### 3.9. Induction of Intracellular ROS/Superoxide Production

ROS induction in A2780 cells after incubation with 4 μM complex (**1**) was studied using a ROS-ID^®^ Total ROS/Superoxide detection kit (Enzo Life Sciences, Farmingdale, NY, USA) according to the manufacturer’s protocol. The cells were seeded in a 96-well cell culture plates at 10^4^ cells per well and cultivated for 24 h, followed by 24 h experimental treatment. Then, the cells were stained with ROS/superoxide probe mixture and incubated at 37 °C in the dark for 60 min. Finally, the fluorescence was measured using a microplate reader Infinite M200Pro (Tecan, Männedorf, Switzerland). The control samples contained 500 µM pyocyanin, and three independent experiments were conducted, each in triplicate.

### 3.10. Mitochondrial Membrane Potential Analysis

The A2780 cells were seeded in 24-well cell culture plates at 5 × 10^4^ cells/well and cultivated for 24 h. The next day, the complex (**1**) was added to a final concentration of 4 µM, and the cells were incubated for another 24 h. After experimental treatment, the cells were washed with PBS, detached by 0.25% trypsin-EDTA (Gibco™), and resuspended in a cell culture medium. The cells were then isolated by centrifugation and resuspended in a staining solution prepared according to the manufacturer’s protocol from a MITO-ID^®^ Membrane Potential Detection kit (Enzo Life Sciences, USA). Finally, the cells were analyzed using a BD FACSVerse flow cytometer (Becton Dickinson, USA). The control was carried out by treatment with 2 µM carbonyl cyanide 3-chlorophenylhydrazone (CCCP). Three independent experiments were conducted, each in duplicate.

### 3.11. Cell Maintenance and Viability Determination

THP1-Blue™ NF-κB cell line (InvivoGen; San Diego, CA, USA) was used to evaluate anti-NF-κB and pro-PPARγ actions of the tested compounds, i.e., complex (**1**) and its precursors [Au(PPh_3_)Cl] and free kinetin, as well as the reference drug *Auranofin* (Merck, Rahway, NJ, USA). The cells were cultivated in RPMI (Roswell Park Memorial Institute) 1640 medium (Biosera; Nuaille, France) supplemented with 10% fetal bovine serum (FBS) and antibiotics (100 U/mL penicillin and 100 mg/mL streptomycin) (both from Merck). All further experiments were performed in serum-free medium. The effect of the test compounds dissolved in DMF on cell viability after 24 h incubation was determined using a Cell Counting Kit-8 (CCK-8; Merck) according to the manufacturer’s instructions, similarly as previously described [18].

### 3.12. NF-κB Activity Determination

The effect of the tested compounds to influence the function of transcription factor NF-κB was determined on lipopolysaccharide (LPS)-challenged THP1-Blue™ NF-κB cells as we described previously [44]. Briefly, the cells were pre-treated by the tested compounds dissolved in DMF at the non-toxic concentration of 100 nM for 1 h. Then, LPS from *E. coli* 0111:B4 (Merck) dissolved in serum-free RPMI 1640 medium (1 µg/mL) was added. After 24 h incubation, the activity of NF-κB was determined as the amount of secreted embryonic alkaline phosphatase using Quanti-Blue™ medium (Invivogen).

### 3.13. Immunocytochemical Analysis of PPARγ Expression and Localisation

THP1-Blue™ NF-κB cells were seeded onto a µ-Slide 8 Well (Ibidi; Gräfelfing, Germany) and differentiated into macrophages by myristate acetate (PMA) at the final concentration of 50 ng/mL, as was described previously [20].

Differentiated macrophages were washed by PBS and covered by fresh serum-free medium for 2 h. Then, the tested compounds were added at the concertation of 100 nM (for complex (**1**), free kinetin, [Au(PPh_3_)Cl], and *Auranofin*), and 1 µM for the control drug pioglitazone (Merck). One hour later, the cells were challenged by LPS for 24 h. Then, the cells were washed by PBS and fixed by 4% paraformaldehyde dissolved in PBS for 10 min. Cell permeabilization and blocking of free binding sites were performed by 0.1% (*v*/*v*) Triton X100 and 5% (*w*/*v*) bovine serum albumin (BSA) dissolved in PBS for 1 h at room temperature. After that, cells were washed by PBS with 0.1% (*v*/*v*) Tween 20 and covered by fresh PBS with 5% BSA and 0.1% Tween 20 containing Alexa Fluor^®^ 546-labelled anti-PPARγ antibody diluted 1:100 (catalogue number sc-7273; Santa Cruz Biotechnology; Dallas, TX, USA) for a further 24 h. Then, cells were washed by PBS with 0.1% Tween 20 and incubated with Hoechst stain diluted 1:2000 for 10 min. The remaining stain was washed out by PBS and covered by fresh PBS for further microscope analysis. The confocal microscope Leica TCS SP8 MP (Leica Microsystems, Mannheim, Germany) was used for obtaining the images for the immunocytochemical analysis of PPARγ expression and localization.

### 3.14. Proteomic Analysis

The differentially treated R2780 cells with the gold complexes [Au(kin)(PPh_3_)] (**1**) and [Au(PPh_3_)Cl], and reference samples kinetin and dimethylformamide (DMF), were harvested and washed with a standardized phosphate buffer, and the cells were counted. An equal number of cells were further processed.

Proteins from the cells were released using a solution consisting of 5% sodium deoxycholate dissolved in 100 mM TEAB (pH 8.0). The cells were disrupted by pipetting and heated at 85 °C for 10 min in a thermomixer. After cooling, the samples were treated by 200 U of nucleic benzoase for 15 min at 25 °C in a thermomixer and finally centrifuged at 20,000× *g* for 10 min at +4 °C. The residual pellet in each sample was removed, and the supernatants were used in solution digestion with commercial trypsin (SoluTrypsin, Merck, USA) as previously described [45]. Each biological sample was processed in four technical replicates.

After digestion, the sodium deoxycholate was removed by using the phase-transfer procedure with ethylacetate [46]. The tryptic peptides were purified on Macro SpinColumns (Harvard Apparatus, Massachusetts, USA) filled with a reversed-phase (C18) according to the manufacturer’s protocol and analyzed by LC-MS/MS using settings adapted from Chamrád et al. [47]. The collected Tims-MS data were processed and searched using MaxQuant software, version 2.1.4.0 [48], with the “TIMS-DDA” instrument parameter setting [49] and the Andromeda engine [50]. Protein identification was conducted against the human protein database (UniProt, reference proteome UP000005640, 80,581 protein sequences, downloaded 11 December 2022). Statistical analysis was carried out using Perseus software, version 2.0.7.0 [51]. The enriched clusters of functional annotation GO terms related to the identified proteins were determined using DAVID Bioinformatics Resources v.2021 [52]. For detailed results of proteomic analyses, please see the data files provided in the Supplementary Materials.

## 4. Conclusions

The title gold(I) complex (**1**) containing the plant hormone kinetin and triphenylphosphine as ligands, [Au(kin)(PPh_3_)], was prepared, thoroughly characterized, and evaluated for its anti-inflammatory and anticancer features at the in vitro level. The results revealed that the complex (**1**) showed significant in vitro cytotoxicity against human cancer cell lines (A2780, A2780R, PC-3, 22Rv1, and THP-1), with IC_50_ ≈ 1–5 μM, which is better than that of a conventional platinum-based drug *Cisplatin* and comparable with a reference gold-based metallotherapeutic *Auranofin*. Its ability to inhibit transcription factor NF-κB did not exceed the effect of *Auranofin*; however, it showed a promising effect on moderation of the inflammatory response through elicitation of peroxisome-proliferator-activated receptor gamma (PPARγ). The cellular effects of the complex (**1**) in A2780 cancer cells were studied in detail by molecular biology methods and were compared with the effects of *Cisplatin* and *Auranofin*. The obtained results revealed that complex (**1**) had the ability to modify the cell cycle of A2780 cells by G2/M and S-phase arrest, showing moderate pro-apoptotic effects; did not influence the processes leading to autophagy; and only slightly impeded the mitochondrial membrane potential of A2780 cells. The proteomic study confirmed its ability to significantly influence the processes of DNA damage/repair, cell division, mitochondrial metabolism, mRNA processing/splicing, and intracellular signal transduction. Although the complex (**1**) did not reveal dual anti-inflammatory action, it showed significant in vitro anticancer effects, and thus it may represent a suitable candidate for forthcoming and deeper biological evaluations.

## Figures and Tables

**Table 1:** Selected bond lengths (Å) and angles (°) for [Au(kin)(PPh_3_)] (**1**) and their comparison with similar Au(I) complexes containing *O*-ethyl-7-deazahypoxanthine (HL1) and *O*-propyl-7-deazahypoxanthine (HL2).

Compound	[Au(kin)(PPh_3_)]		[Au(L1)(PPh_3_)] ^a^	[Au(L2)(PPh_3_)] ^b^
Selected bond lengths (Å)	Au-N(9)	2.046(3)	2.046(3)	2.042(3)
	Au-P	2.2379(10)	2.2344(9)	2.2356(8)
Selected angle (°)	N(9)-Au-P	178.64(9)	173.73(10)	175.65(8)

^a^ reference [19], ^b^ reference [20].

**Table 2:** The results of in vitro cytotoxicity against selected human cancer cell lines (A2780, A2780R, PC-3, 22Rv, THP-1, and LS180) and healthy (MRC5) cells. IC_50_ values are given in µM together with the standard deviations, and they are based on triplicate experiments. The abbreviation n.d. means that the value has not been determined.

Compound	Human Cell Lines						
	A2780	A2780R	PC-3	22Rv1	THP-1	LS180	MRC5
[Au(kin)(PPh_3_)] (**1**)	2.9 ± 0.7	5.0 ± 1.5	4.8 ± 1.3	3.7 ± 0.5	1.1 ± 0.2	>10	>10
Kinetin	>25	>25	>25	n.d.	n.d.	n.d.	>25
[Au(PPh_3_)Cl]	3.5 ± 0.5	5.2 ± 0.2	9.9 ± 0.4	n.d.	n.d.	n.d.	17.0 ± 0.5
*Auranofin*	3.4 ± 0.9	3.4 ± 0.6	3.7 ± 0.7	n.d.	n.d.	n.d.	3.78 ± 0.01
*Cisplatin*	17.2 ± 0.9	>50	>50	28.2 ± 4.2	n.d.	>50	>50

**Table 3:** The results of time-dependent in vitro cytotoxicity against the A2780 cell line.

Compound	24 h	48 h	72 h
[Au(kin)(PPh_3_)] (**1**)	2.9 ± 0.7	3.0 ± 0.3	3.0 ± 0.6
[Au(PPh_3_)Cl]	3.5 ± 0.5	3.2 ± 0.2	3.1 ± 0.3
*Auranofin*	3.4 ± 0.9	2.1 ± 0.4	2.0 ± 0.1
*Cisplatin*	17.2 ± 0.9	12.4 ± 1.8	5.8 ± 1.8

**Table 4:** Crystal data and structure refinement for [Au(kin)(PPh_3_)] (**1**).

Empirical formula Formula weight Temperature Wavelength Crystal system Space group Unit cell dimensions Volume *Z* Density (calculated) Absorption coefficient *F*(000) Crystal size θ range for data collection Index ranges Reflections collected Independent reflections Completeness to θ = 25.242° Absorption correction Refinement method Data/restraints/parameters Goodness-of-fit on *F^2^* Final *R* indices [*I* > 2σ(*I*)] *R* indices (all data) Extinction coefficient	C_28_ H_23_ Au N_5_ O P 673.45 293(2) K 0.71073 Å Triclinic P-1 a = 10.050(4) Å, α = 88.980(12)° b = 11.779(5) Å, β = 79.919(15)° c = 13.847(5) Å, γ = 70.122(19)° 1516.2(10) Å^3^ 2 1.475 g/cm^3^ 4.930 mm^−1^ 656 0.08 × 0.08 × 0.06 mm 1.84 to 27.59°. −13 ≤ *h* ≤ 13, −15≤ ≤ *k* ≤ 15, −17 ≤ *l* ≤17 41,317 6986 [*R*(int) = 0.0744] 100.0% Semi-empirical from equivalents Full-matrix least-squares on *F*^2^ 6986/0/325 1.044 *R*1 = 0.0313, *wR*2 = 0.0546 *R*1 = 0.0539, *wR*2 = 0.0602 n/a

## Data Availability

The Cambridge Crystallographic Database contains the supplementary crystallographic data for the [Au(kin)(PPh_3_)] complex (**1**), CCDC deposition number: 2231975. These data can be obtained free of charge via www.ccdc.cam.ac.uk/conts/retrieving.html, accessed on 28 April 2022 (or from the Cambridge Crystallographic Data Centre, 12 Union Road, Cambridge CB21EZ, UK; fax: (+44) 1223-336-033; or de-posit@ccdc.cam.ac.uk).

## Supplementary Materials

The following supporting information can be downloaded at https://www.mdpi.com/article/10.3390/ijms24032293/s1.
