# Supplementary material for: The Gold(I) Complex with Plant Hormone Kinetin Shows Promising In Vitro Anticancer and PPARγ Properties
Source: Int J Mol Sci. 2023 Jan 24;24(3):2293. doi: 10.3390/ijms24032293 (PMC9916778; doi:10.3390/ijms24032293)
Supplement: Supplementary file 1 [file ijms-24-02293-s001.zip › ijms-2146245-supplementary.pdf]

Supplementary Materials

**Table of Contents**	**Page**
**Fig. S1** Schematic representations of structures of [Au(kin)(PPh_3)] (**1**) (*left*), free kinetin (Hkin) (*middle*) and free triphenylphosphine (PPh_3) (*right*) together with the atoms labelling for interpretation of NMR data.	S3
**Fig. S2** The IR spectra of [Au(kin)(PPh_3)] (**1**) and free kinetin.	S3
**Fig. S3.** The ESI-MS spectrum of (**1**) measured in MeOH solution in positive ionization mode.	S4
**Fig. S4** The TG/DSC curves characterizing the thermal decomposition of complex (**1**). The most significant mass changes in TG and peak temperatures in DSC record are noted.	S4
**Fig. S5** The N–H···N hydrogen bonds (cyan dashed lines) connecting two individual molecules of (**1**).	S5
**Fig. S6** Part of crystal structure of (1) showing the C–H···N, C–H···C and C···C non-covalent contacts (red dotted lines) connecting individual molecules of (**1**) into a 3D structure.	S5
**Table S1** Hydrogen bond for [Au(kin)(PPh_3)] (**1**) [Å and °].	S6
**Fig. S7** The effects of (**1**), the starting complex [Au(PPh_3)Cl] and reference drugs Auranofin and Cisplatin on intracellular ROS levels in A2780 cells after 24 h of incubation with half-cytotoxic concentrations of the compounds in the presence (Pyocyanin+) and absence (Pyocyanin-) of the well-known oxidative stress inducer pyocyanin. The statistical significance was considered at the following levels: * $p<0.05$, ** $p<0.01$, *** $p<0.001$ with respect to the untreated control group.	S6
**Fig. S8** The effects of (**1**), the starting complex [Au(PPh_3)Cl] and reference drugs Auranofin and Cisplatin on intracellular superoxide levels in A2780 cells after 24 h of incubation with half-cytotoxic concentrations of the compounds in the presence (Pyocyanin+) and absence (Pyocyanin-) of the well-known oxidative stress inducer pyocyanin. The statistical significance was considered at the following levels: * $p<0.05$, ** $p<0.01$, *** $p<0.001$ with respect to the untreated control group.	S7
**Table S2** Determination of cytotoxic effect of the tested compounds expressed as IC_{50} values for 24 h treatment. Data are shown as mean ± SEM.	S7
**Fig. S9** The effect of the tested compounds on the amount and intracellular localisation of PPARγ in THP1-Blue™ NF-κB macrophages after 1 h pre-treatment.	S8

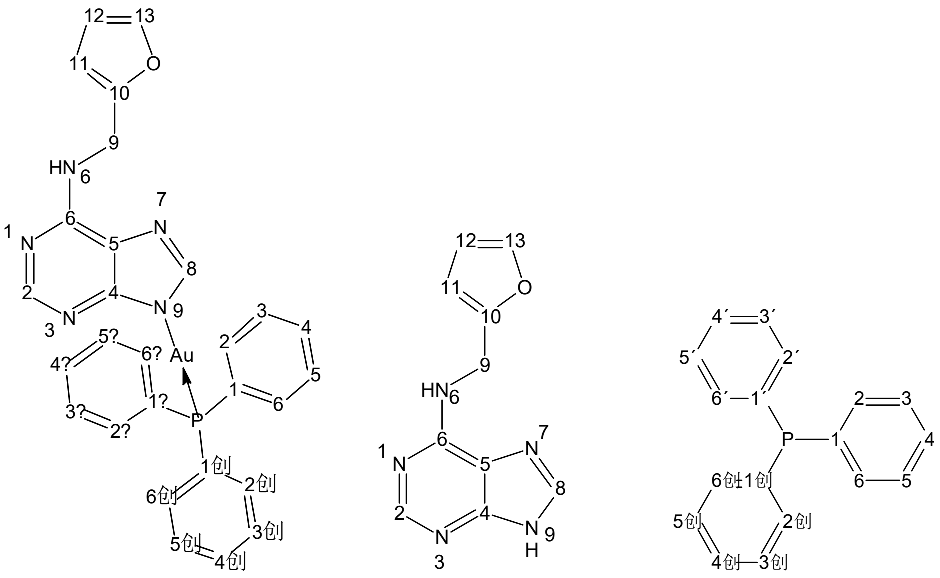

Fig. S1 Schematic representations of structures of [Au(kin)(PPh3)] (**1**) (*left*), free kinetin (Hkin) (*middle*) and free triphenylphosphine (PPh3) (*right*) together with the atoms labelling for interpretation of NMR data.

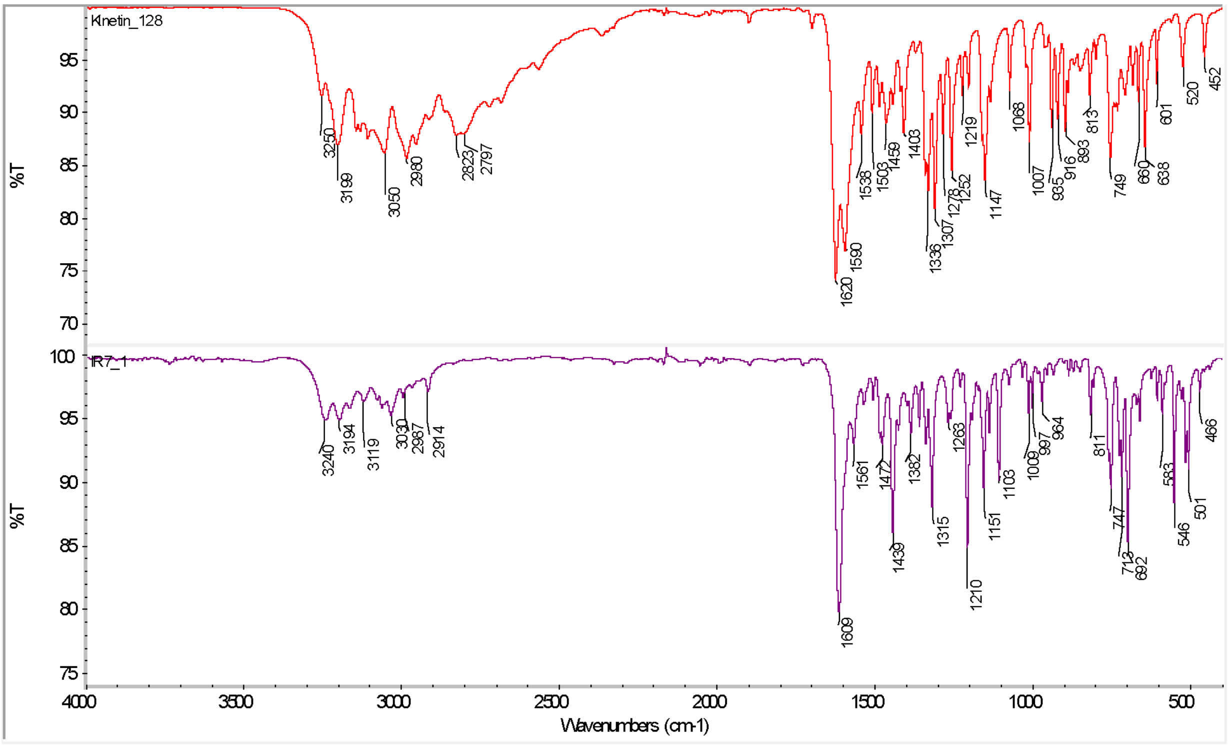

Fig. S2 The IR spectra of [Au(kin)(PPh_3)] (**1**) (*down*) and free kinetin (*up*).

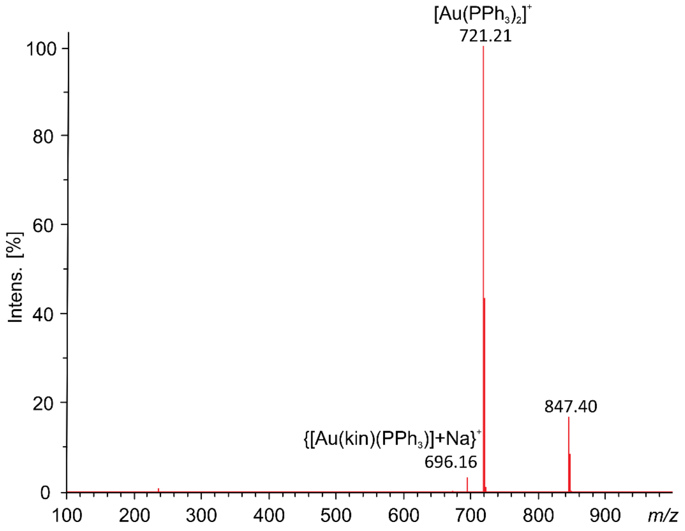

Fig. S3. The ESI-MS spectrum of (**1**) measured in MeOH solution in positive ionization mode.

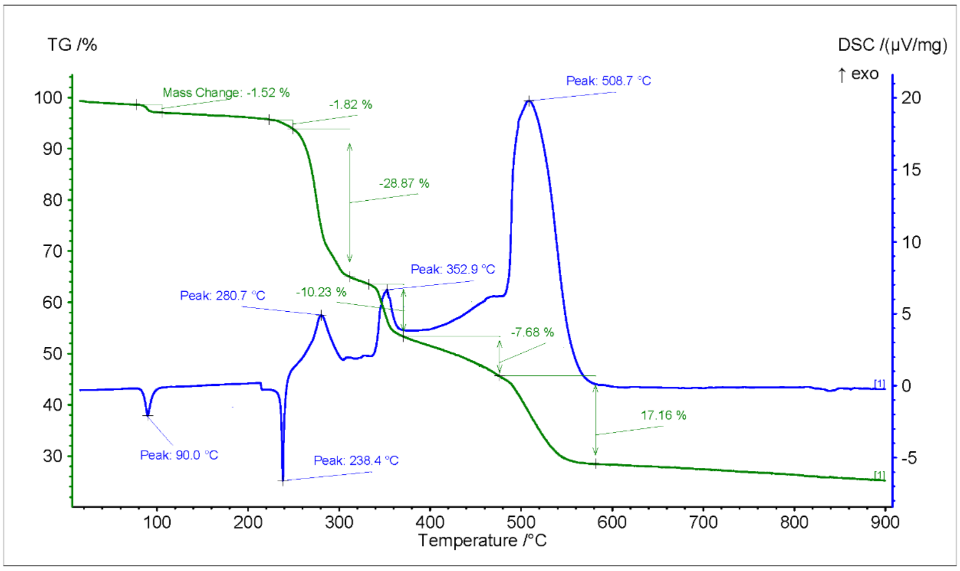

Fig. S4 The TG/DSC curves characterizing the thermal decomposition of complex (**1**). The most significant mass changes in TG and peak temperatures in DSC record are noted.

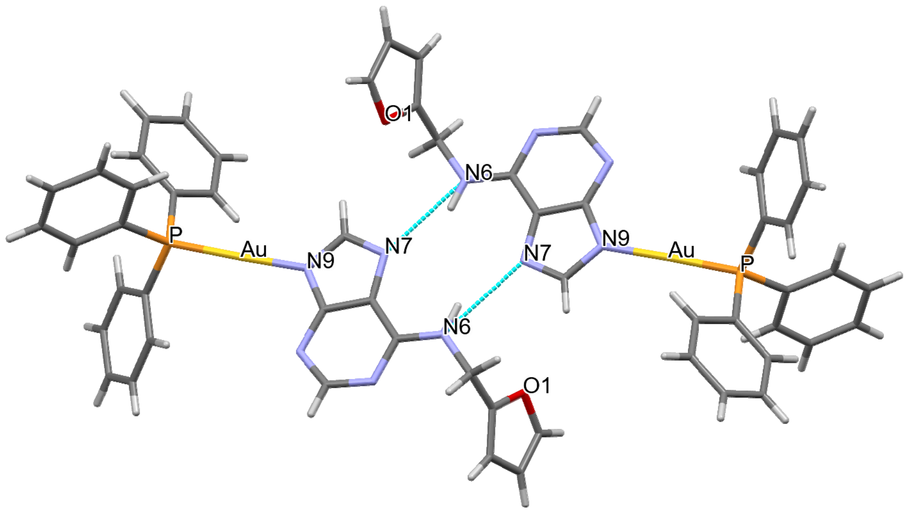

Fig. S5 The N–H···N hydrogen bonds (cyan dashed lines) connecting two individual molecules of (**1**).

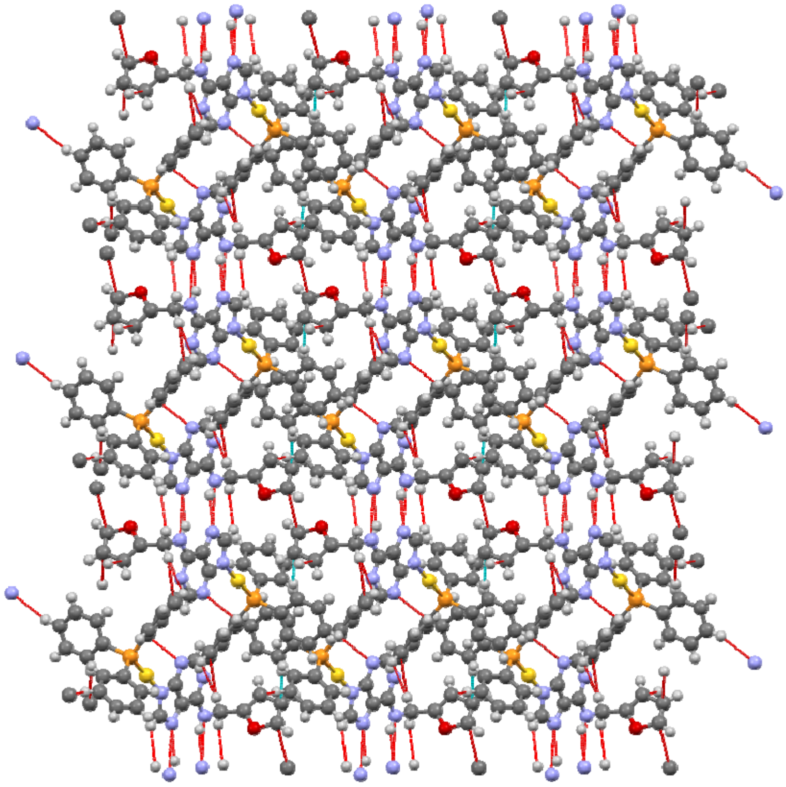

Fig. S6 Part of crystal structure of (**1**) showing the C–H···N, C–H···C and C···C non-covalent contacts (red dotted lines) connecting individual molecules of (**1**) into a 3D structure.

Table S1 Hydrogen bond for [Au(kin)(PPh_3)] (**1**) [Å and °].

D–H···A	d(D–H)	d(H···A)	d(D···A)	<(DHA)
N(6)–H(6A)...N(7)#1	0.86	2.20	2.988(5)	151.4

Symmetry transformation used to generate equivalent atoms: #1 -x, -y+1, -z

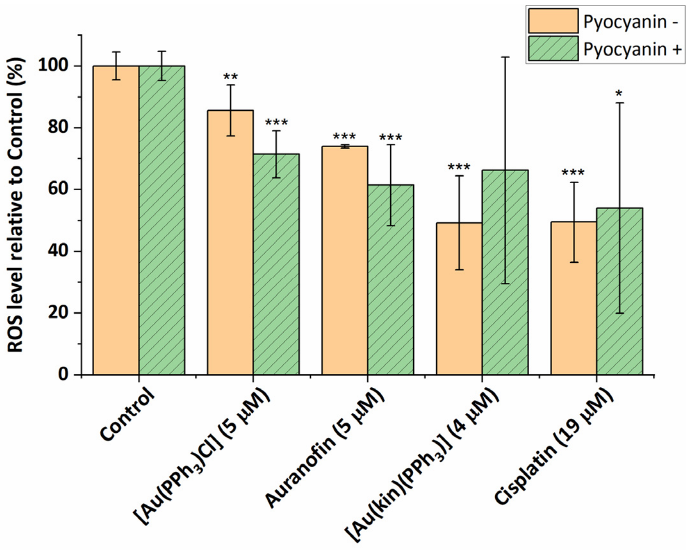

Fig. S7 The effects of (**1**), the starting complex [Au(PPh_3)Cl] and reference drugs *Auranofin* and *Cisplatin* on intracellular ROS levels in A2780 cells after 24 h of incubation with half-cytotoxic concentrations of the compounds in the presence (Pyocyanin+) and absence (Pyocyanin-) of the well-known oxidative stress inducer pyocyanin. The statistical significance was considered at the following levels: * $p<0.05$, ** $p<0.01$, *** $p<0.001$ with respect to the untreated control group.

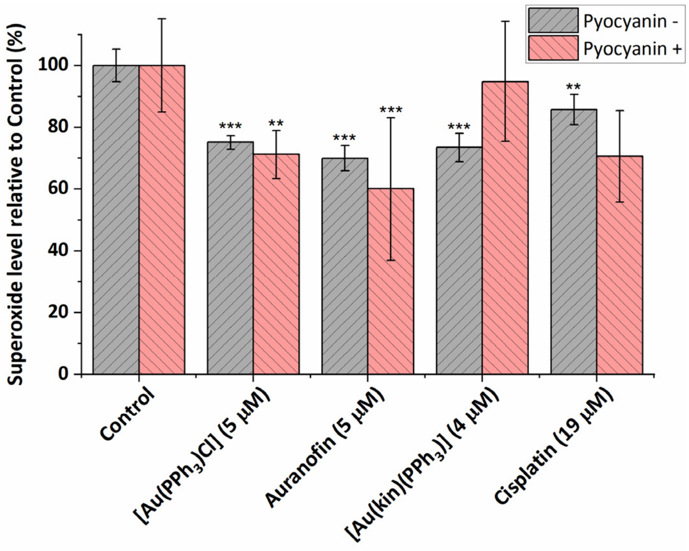

Fig. S8 The effects of (**1**), the starting complex [Au(PPh$_3$)Cl] and reference drugs *Auranofin* and *Cisplatin* on intracellular superoxide levels in A2780 cells after 24 h of incubation with half-cytotoxic concentrations of the compounds in the presence (Pyocyanin+) and absence (Pyocyanin-) of the well-known oxidative stress inducer pyocyanin. The statistical significance was considered at the following levels: ** p<0.01, *** p<0.001 with respect to the untreated control group.

Table S2 Determination of cytotoxic effect of the tested compounds expressed as IC$_{50}$ values for 24 h treatment. Data are shown as mean ± SEM.

	IC$_{50}$ ± SEM (95% confidence interval)
[Au(kin)(PPh$_3$)] (**1**)	1.0 ± 1.0 µM (0.9 – 1.1 µM)
Auranofin	0.5 ± 1.1 µM (0.4 – 0.6 µM)
[Au(PPh$_3$)Cl]	1.1 ± 1.1 µM (1.0 – 1.2 µM)
Kinetin	> 10 µM

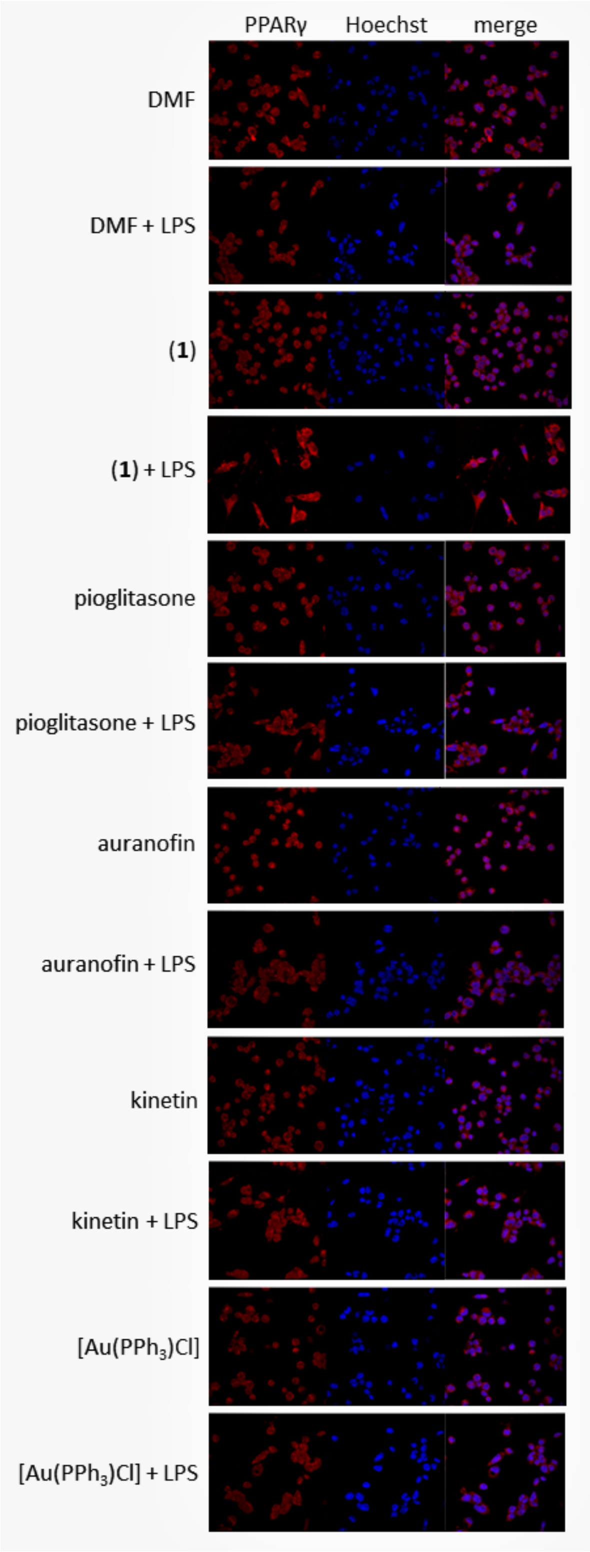

Fig. S9 The effect of the tested compounds on the amount and intracellular localisation of PPARγ in THP1-Blue™ NF-κB macrophages after 1 h pre-treatment.
